# Assessment of Physiological, Biochemical, Hormonal, and Sexual Health Parameters in Patients Using Protein Supplements and Anabolic-Androgenic Steroids: A Cross-Sectional Study

**DOI:** 10.7759/cureus.117790

**Published:** 2026-10-11

**Authors:** Amit S Mutha, Amit S Beldar, Kamagonda Jalikar, Ajinkya M Bavlecha, Kunal Verma, Roshan Pawar, Nancy Bansal

**Affiliations:** 1 Department of Pharmacology, Government Medical College, Gokuldas Tejpal Hospital and Cama Albless Hospital, Mumbai, IND; 2 Department of Pharmacology, Dr. Vasantrao Pawar Medical College, Hospital and Research Centre, Nashik, IND; 3 Department of Pharmacology, SDM College of Medical Sciences and Hospital, Shri Dharmasthala Manjunatheshwara University, Dharwad, IND

**Keywords:** anabolic-androgenic steroids, gym supplements, hematological parameters, hormonal imbalances, sexual dysfunction

## Abstract

Background

Protein supplements (PS) and anabolic-androgenic steroids (AAS) are used to enhance physique and performance and have been associated with alterations in physiological, biochemical, hormonal, and sexual health parameters. This study evaluated the impact of PS and AAS on hematological, biochemical, hormonal, and sexual health indices.

Materials and methods

A cross-sectional study was conducted among male patients of reproductive age presenting with sexual complaints to an andrology clinic. Of 1567 screened individuals, 112 users of PS or AAS were enrolled and grouped as PS only (n = 62), AAS only (n = 18), and PS+AAS (n = 32). A comparison group of 50 volunteers without sexual complaints or PS or AAS use was included. Participants were evaluated for hematological, liver, and renal functions and hormonal profiles using available investigation records. Sexual complaints were documented. Data were analyzed using appropriate statistical tests, wherever applicable (p<0.05).

Results

Abnormal findings were least frequent in PS users and most pronounced in PS+AAS users. Elevated aspartate aminotransferase (AST) occurred in 44.4% of AAS users and 46.8% of PS+AAS users. Reduced luteinizing hormone (LH) was seen in 61.1% and 68.7% of AAS and PS+AAS users, reduced follicle-stimulating hormone (FSH) in 50.0% and 53.1%, and elevated estradiol in 38.8% and 40.6%, respectively. Testosterone showed a biphasic pattern. Gynecomastia, altered libido, testicular atrophy, reduced ejaculate volume, and other sexual-health complaints were more frequent in AAS-containing groups.

Conclusion

AAS use, particularly with concurrent PS use, was associated with biochemical, hormonal, and sexual health abnormalities, while PS-only users showed fewer abnormalities. These findings underscore the need for awareness, regulatory oversight, and screening among supplement users.

## Introduction

Ergogenic aids have been used for centuries and include nutritional, pharmacological, physiological, psychological, and mechanical approaches [1-4]. Sports supplements are orally ingested products containing dietary ingredients derived from minerals, plants, extracted tissues, or chemical synthesis/processing [5], available as powders, drinks, pills/capsules, gels, and bars [6].

Protein supplements (PS) are protein-containing nutritional supplements used for fitness or performance purposes, has increased worldwide beyond elite athletes, particularly among male commercial gym exercisers, often with creatine and amino acids [7]. Testosterone and its derivatives have been used for decades. Since isolation in 1935, multiple derivatives have been synthesized, labelled anabolic-androgenic steroids (AAS), and increasingly abused among recreational athletes and also female professionals [8]. PS and AAS are used to enhance muscle mass, strength, and libido [9,10], for performance/fitness, weight control, energy, special nutrient demands, immunity, disease prevention/treatment, alertness, stress reduction, and psychological well-being [11].

Despite benefits, harms are reported. High protein intake may increase urea/albumin/calcium/oxalate excretion, cause renal hyperfiltration, worsen chronic kidney disease risk, and promote renal stones via acid excretion and reduced renal pH [12]. Hepatic effects remain understudied. Animal studies show hepatic dysfunction with raised transaminases and hepatocyte injury [13], and evidence remains controversial [14]. Nonhormonal supplements may also be adulterated with undeclared AAS (~14.8%) [15].

Despite increasing use, comparative real-world data on hematological, biochemical, hormonal, and sexual-health parameters among PS users, AAS users, and combined users remain limited. The objective of this study was to compare these parameters across PS users, AAS users, combined PS+AAS users, and a comparison group.

## Materials and methods

A cross-sectional observational study was conducted at the Grant Govt. Medical College and Sir J.J. Group of Hospitals, Mumbai, India, among patients presenting to the outpatient department with sexual complaints, in whom a current clinical assessment was performed and relevant prior clinical history and investigation records were reviewed. The study included male patients aged between 18 and 55 years who presented to the outpatient department of the andrology clinic with sexual complaints and had a history of consumption of PS or abuse of AAS or both. Ethics Committee approval was obtained from the Grant Govt. Medical College and Sir J.J. Group of Hospitals (No. IEC/Pharm/RP/113/Mar/2018, dated 01/03/2018). Patients were included in the study if they presented to the outpatient department with sexual health complaints, including gynecomastia, erectile dysfunction (ED), premature or delayed ejaculation, anorgasmia, decreased libido, testicular atrophy/reduced testicular volume, reduced ejaculate volume, or infertility, and had a history of PS and/or AAS use, only after obtaining written informed consent. Patients were excluded from the study if they were diagnosed with any psychiatric illness or were on any prescription medication capable of altering the sexual health of the individuals. Patients were also excluded if they presented with a previous history of endocrine disorders, previously diagnosed infertility, chronic systemic illness, or were unaware of the substance they were consuming. 

A total of 1567 patients were screened, of whom 112 met the eligibility criteria and were included in the exposure groups. These comprised 62 PS-only users, 18 AAS-only users, and 32 combined PS+AAS users. An additional comparison group of 50 volunteers without sexual complaints and without PS or AAS use was included, giving a total study population of 162 participants. No formal a priori sample-size calculation was performed. Convenience sampling was used, with eligible participants presenting during the study period included in the study. The comparison group was not formally matched to the exposure groups for variables such as age, BMI, or lifestyle factors.

Participants were enrolled and clinically assessed at the time of presentation, and relevant prior clinical history and available investigation records were reviewed. The blood tests assessed hematological parameters like hemoglobin, hematocrit, red blood cell (RBC) count, white blood cell (WBC) count, and platelet count. Liver function tests assessed alanine aminotransferase (ALT), aspartate aminotransferase (AST), alkaline phosphatase (ALP), gamma-glutamyl transferase (GGT), and total bilirubin. Additionally, renal function tests (RFTs), which included creatinine, blood urea, and uric acid, were also evaluated. Finally, a hormone profile consisting of total testosterone, luteinizing hormone (LH), follicle-stimulating hormone (FSH), and estradiol was also analyzed. Laboratory values were interpreted using standard reference ranges for the respective hematological, biochemical, renal, and hormonal parameters. Patients were also evaluated for sexual health complaints. The symptoms and signs assessed included gynecomastia, ED, premature or delayed ejaculation, anorgasmia, decreased libido, testicular atrophy/reduced testicular volume, reduced ejaculate volume, and infertility.

The data were collected in a case record form, compiled into a Microsoft Excel (Microsoft Corporation, Redmond, Washington, United States) spreadsheet, and analyzed using IBM SPSS Statistics for Windows, Version 26 (Released 2018; IBM Corp., Armonk, New York, United States). The results were studied for each group and represented in simple percentages, tables, and graphs, wherever necessary. Normality of continuous variables was assessed using the Kolmogorov-Smirnov test. Parametric or non-parametric tests were selected according to the data distribution. One-way ANOVA followed by Tukey’s post-hoc test was used for normally distributed data, while the Kruskal-Wallis test followed by Dunn’s post-hoc test was used for non-normally distributed data, wherever applicable. Statistical significance was set at p<0.05.

## Results

A total of 162 participants were included in the study. Of these, 112 comprised the exposure groups: 62 PS-only users (Group 1), 18 AAS-only users (Group 2), and 32 combined PS+AAS users (Group 3). The comparison group comprised 50 participants without sexual complaints and without PS or AAS use. The mean ages were 34.12 ± 7.21 years in the comparison group, 35.34 ± 5.45 years in Group 1, 41.33 ± 13.21 years in Group 2, and 39.56 ± 12.31 years in Group 3. There was no statistically significant difference in mean age among the four groups (p = 0.0833), as presented in Table 1.

**Table 1:** Baseline characteristics of groups Values are expressed as mean ± SD. Statistical analysis: one-way ANOVA. A p-value <0.05 was considered statistically significant. PS: protein supplement; AAS: anabolic-androgenic steroid; CI: confidence interval

Group	Exposure	n	Mean age ± SD	95% CI	p-value
Comparison (N)	No PS/AAS	50	34.12 ± 7.21	32.07–36.17	0.0833
Group 1 (G1)	PS only	62	35.34 ± 5.45	33.96–36.72	
Group 2 (G2)	AAS only	18	41.33 ± 13.21	34.76–47.90	
Group 3 (G3)	PS+AAS	32	39.56 ± 12.31	35.12–44.00	

Overall, Group 1 showed the fewest abnormalities, while Group 3 showed the greatest number of abnormal findings across most parameter categories. The AAS-containing groups showed more prominent hormonal abnormalities than the PS-only group. Detailed group-wise findings are presented below.

In the hematological profile, patients in all three groups showed an increase in hemoglobin, hematocrit, and RBC count. Although an alarming 40.6% of patients showed an increase in RBC count in Group 3, only 14.5% were affected in Group 1. In Group 2, five (27.7%) patients showed an increase in RBC count and hematocrit value. The increase in hematocrit value was not seen in as many patients in Group 1 (11.3% of patients). Additionally, no patients in Group 1 showed any increase or decrease in WBC or platelet count. This differed in Groups 2 and 3. In Group 2, one (5.5%) patient showed an increase in WBC and platelet counts, while in Group 3, two (6.2%) and one (3.1%) patients showed an increase in WBC and platelet counts, respectively. Figure 1 shows the percentage of patients affected in each group for each hematological parameter.

**Figure 1:**
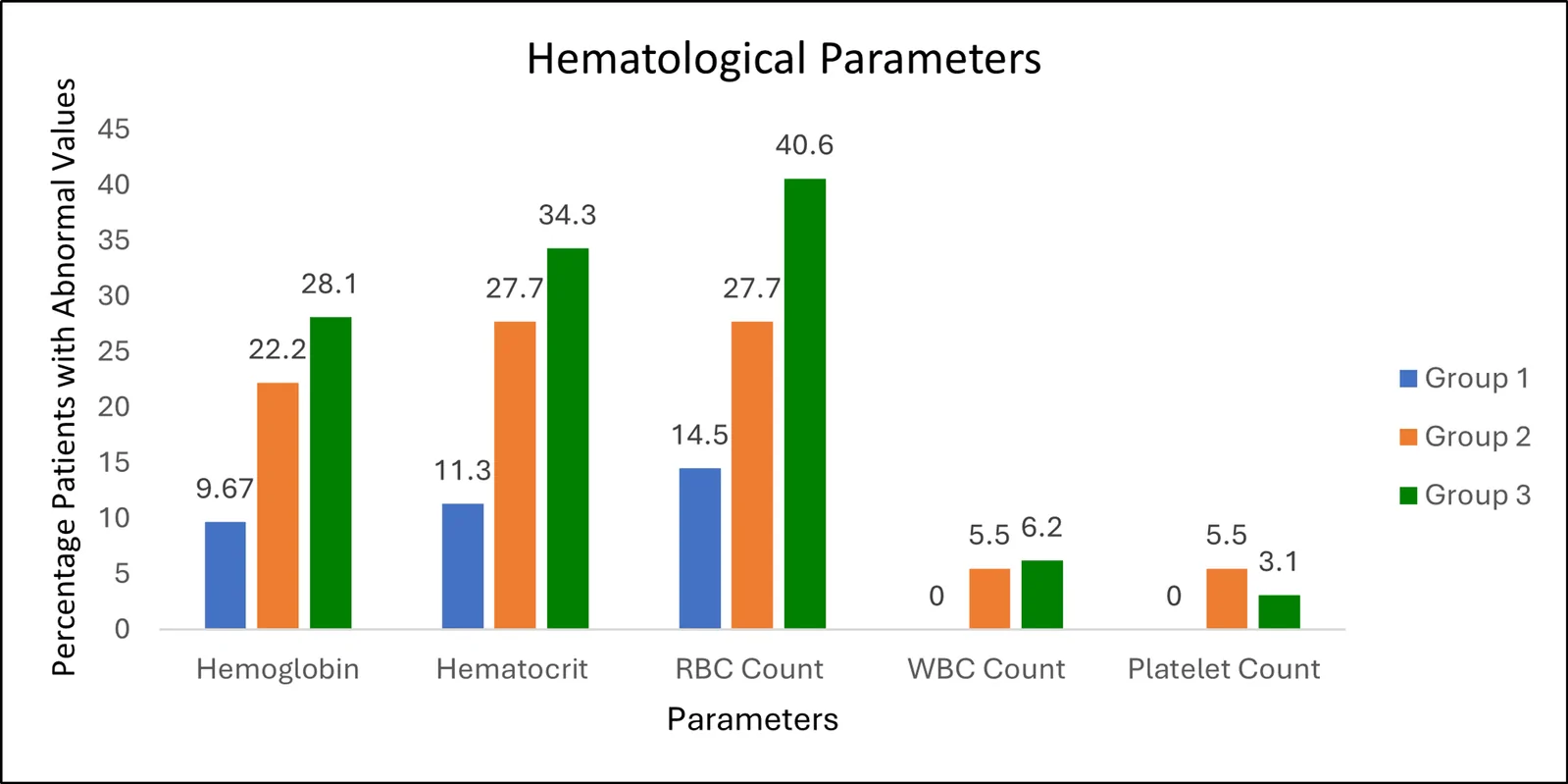
Percentage of affected patients in each group for hematological parameters Group 1: protein supplement (PS) users; Group 2: anabolic-androgenic steroid (AAS) users; Group 3: combined PS and AAS users. Data are presented as the percentage (%) of patients showing abnormal values. This figure is intended as a descriptive representation of the percentage of affected patients across the three exposure groups.

The liver function tests showed a similar pattern, with the most patients affected in Group 3 and the fewest in Group 1. In Group 1, three (4.8%) patients showed an increase in AST and ALT levels. Whereas these values were found to be deranged in significantly more patients in Groups 2 and 3. In Group 2, eight (44.4%) patients showed an increase in AST values, and four (22.2%) patients showed an increase in ALT values. Group 3, with 15 (46.8%) and 10 (31.2%) patients showing an increase in the AST and ALT levels, respectively, had the maximum number of patients affected. In Group 1, no patients showed any derangement in ALP, GGT, or total bilirubin values. However, this was not the same for Groups 2 and 3. In Groups 2 and 3, two (11.1%) and four (12.5%) patients showed increased ALP levels, respectively. The increment in GGT and total bilirubin was found to be present in only one patient in Group 2 and two patients in Group 3. Figure 2 shows the percentage of patients affected in each group for LFT parameters.

**Figure 2:**
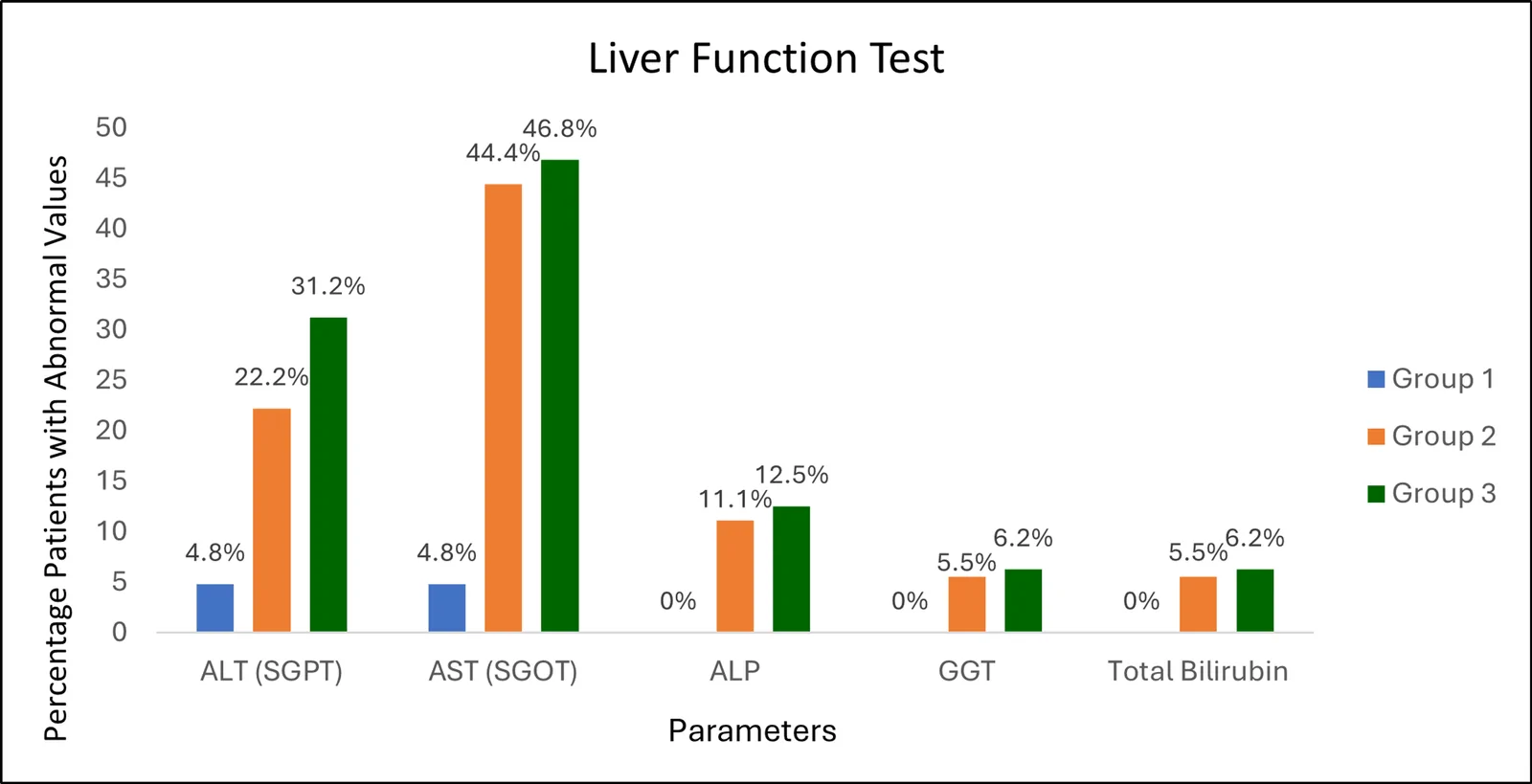
Percentage of affected patients in each group for hepatological parameters Group 1: protein supplement (PS) users; Group 2: anabolic-androgenic steroid (AAS) users; Group 3: combined PS and AAS users. Data are presented as the percentage (%) of patients showing abnormal values. This figure is intended as a descriptive representation of the percentage of affected patients across the three exposure groups. AST: aspartate aminotransferase; ALT: alanine aminotransferase; ALP: alkaline phosphatase; GGT: gamma-glutamyl transferase

Amongst the renal function parameters, the maximum number of patients showed an increase in uric acid levels in 12 (19.3%) patients. Creatinine and blood urea levels were increased in three (4.8%) and nine (14.5%) patients, respectively. Groups 2 and 3 showed similar results. Both groups showed the maximum number of patients with increased uric acid levels. Creatinine was found elevated in two (11.1%) and five (15.6%) patients in Groups 2 and 3, respectively. Additionally, blood urea levels were found to be elevated in all three groups, with the maximum number of patients affected in Group 3, followed by Group 2, and the minimum affected in Group 1. Figure 3 shows the percentage of patients affected in each group for each RFT parameter.

**Figure 3:**
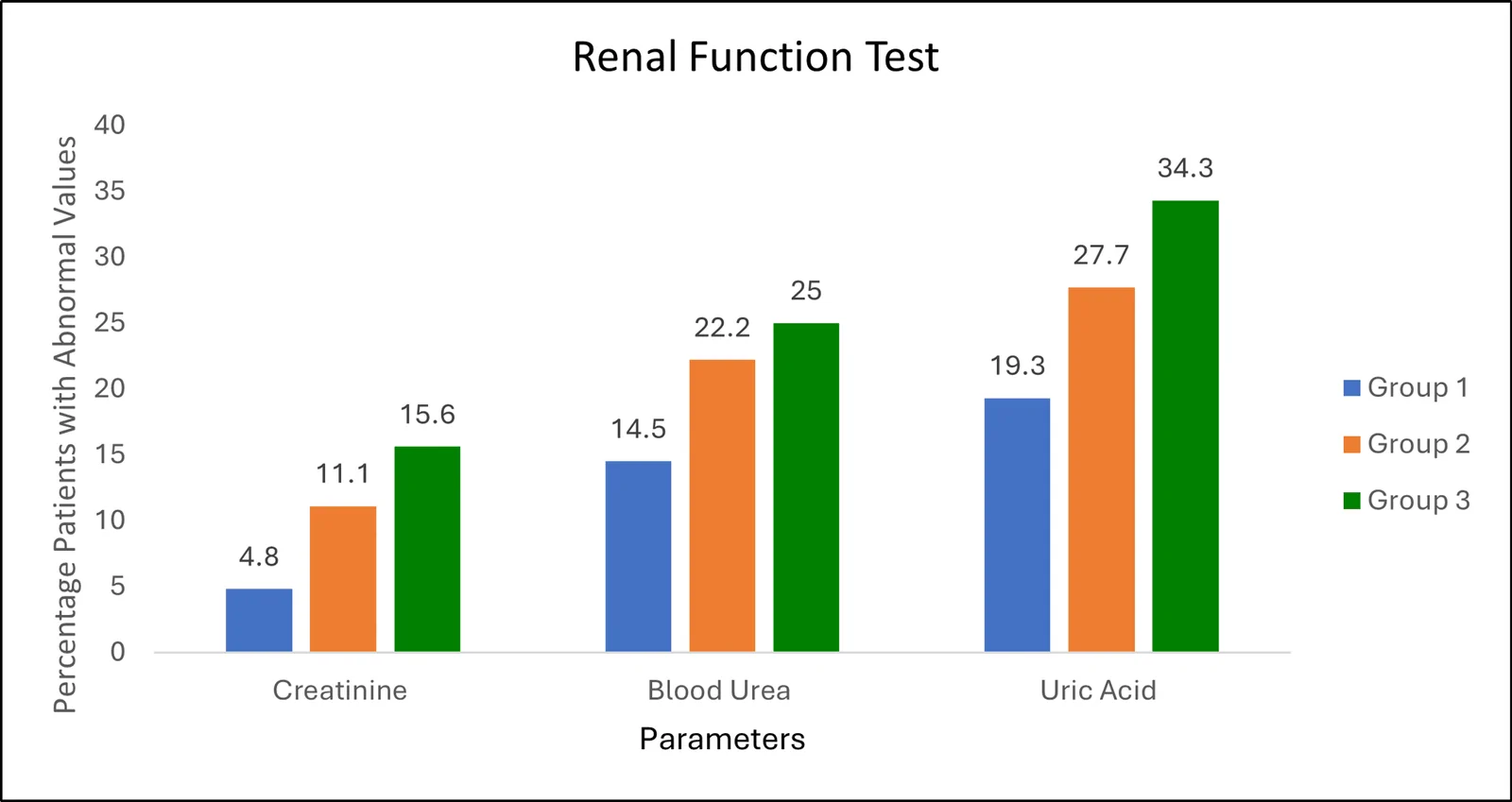
Percentage of affected patients in each group for renal parameters Group 1: protein supplement (PS) users; Group 2: anabolic-androgenic steroid (AAS) users; Group 3: combined PS and AAS users. Data are presented as the percentage (%) of patients showing abnormal values. This figure is intended as a descriptive representation of the percentage of affected patients across the three exposure groups.

The hormonal profile revealed that groups using AAS, which are Groups 2 and 3, had elevated levels of estradiol and decreased levels of FSH and LH. Estradiol was found to be elevated in 38.8% and 40.6% of patients in Groups 2 and 3, respectively. The maximum number of patients in groups using AAS showed decreased levels of LH (61.1% of patients in Group 2 and 68.7% of patients in Group 3). FSH was found to be decreased in 50% of patients in Group 2 and in 53.1% of patients in Group 3. Total testosterone levels in Groups 2 and 3 were found to be elevated in some patients, while others (those who discontinued supplements) showed lower than normal levels. In Group 2, 50% of patients had total testosterone levels below the normal range, while 38.8% had levels above the normal range. Similarly, in Group 3, 43.75% had elevated whereas 53.1% of patients showed reduced levels of testosterone beyond the normal range, respectively. In Group 1, no patients showed any derangements in the hormonal profile beyond the normal range.

Although the hormone levels were not beyond the normal range in Group 1, they were found to be at the extremes in a few patients, mimicking the pattern of Groups 2 and 3 (i.e., estradiol was found to be at the upper extremes, while FSH and LH were found to be towards the lower extremes of the normal range). Figure 4 shows the percentage of patients affected in each group for each hormone profile parameter.

**Figure 4:**
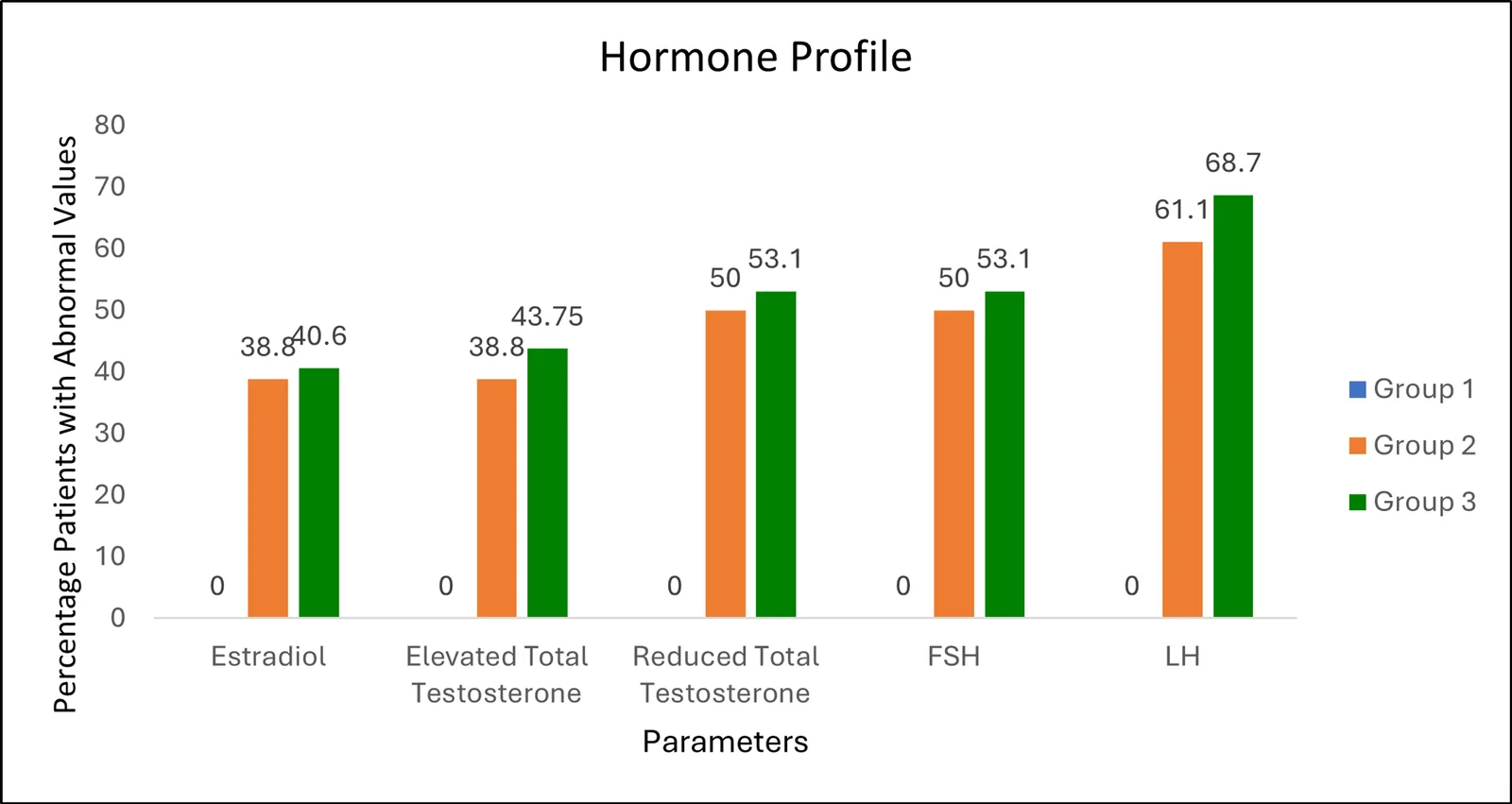
Percentage of affected patients in each group for hormonal parameters Group 1: protein supplement (PS) users; Group 2: anabolic-androgenic steroid (AAS) users; Group 3: combined PS and AAS users. Data are presented as the percentage (%) of patients showing abnormal values. This figure is intended as a descriptive representation of the percentage of affected patients across the three exposure groups. LH: luteinizing hormone; FSH: follicle-stimulating hormone

Sexual complaints included gynecomastia, ED, premature and delayed ejaculation, anorgasmia, decreased and increased libido, testicular atrophy, reduced ejaculate volume, and infertility. These complaints, again, when compared amongst all groups, were found to be maximum in patients belonging to Group 3. Group 1 did not show any complaints of premature ejaculation, anorgasmia, testicular atrophy, or reduced ejaculate volume. Complaints like gynaecomastia, testicular atrophy, decreased libido, and infertility were found to be high in Groups 2 and 3. Figure 5 shows the percentage of people presenting with each complaint amongst all three groups.

**Figure 5:**
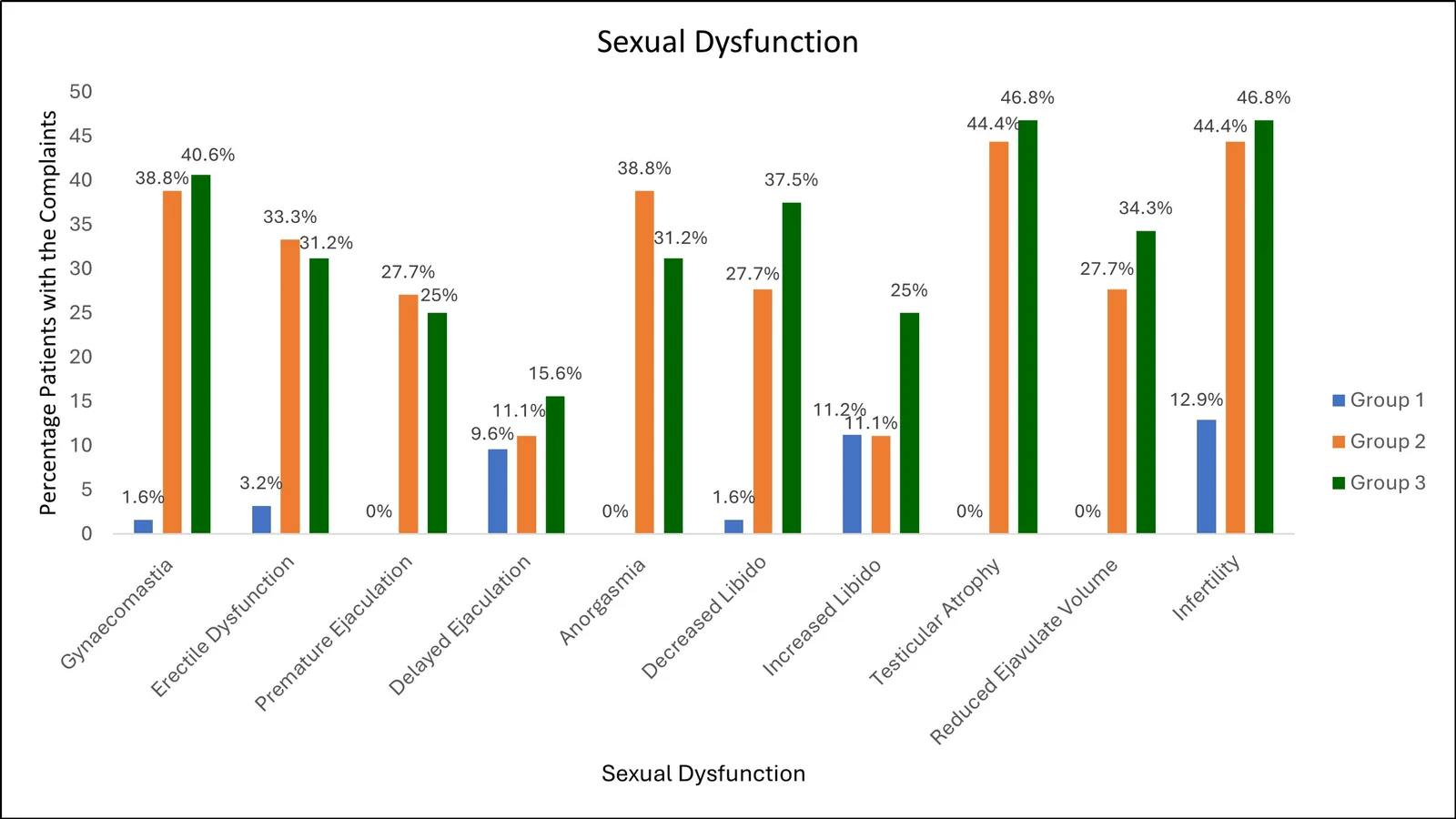
Percentage of patients presenting with sexual dysfunction complaints in each group Group 1: protein supplement (PS) users; Group 2: anabolic-androgenic steroid (AAS) users; Group 3: combined PS and AAS users. Data are presented as the percentage (%) of patients reporting each sexual health complaint. This figure is intended as a descriptive representation of the percentage of patients reporting each sexual health complaint across the three exposure groups.

On comparing all biochemical parameters with normal participants, Group 1 (PS users) remains largely within normal physiological limits, Group 2 (AAS users) shows moderate but distinct deviations, and Group 3 (PS+AAS users) demonstrates the most pronounced alterations.

As shown in Table 2, there were significant differences in the hematological profile, which were mainly observed in hematocrit and RBC count. Hematocrit increased progressively from the Normal group through Groups G1, G2, and G3, with significant differences between G1 and both G2 and G3, as well as between the Normal group and both steroid-using groups. RBC values showed an even greater change, with G2 and G3 significantly higher than both the Normal group and G1. G3 also differed from G2, suggesting a dose-dependent erythropoietic response. Hemoglobin, WBC counts, and platelet counts did not differ significantly across groups, indicating that AAS selectively stimulated erythropoiesis without affecting leukopoiesis or thrombopoiesis.

**Table 2:** Comparison of groups for hematological parameters N = 50, G1 = 62, G2 = 18, G3 = 32; values expressed as mean ± standard deviation (SD); statistical significance was set at p < 0.05. Superscript letters represent a compact letter display (CLD) based on Tukey’s post-hoc test. Within each parameter row, groups sharing at least one superscript letter are not significantly different, whereas groups with no superscript letter in common are significantly different. Letters are assigned independently for each parameter and do not correspond to fixed study groups. Statistical analysis: one-way ANOVA followed by Tukey’s post-hoc test.

Parameter	Reference Range	Normal (Mean±SD)	G1 (Mean±SD)	G2 (Mean±SD)	G3 (Mean±SD)	F (p-value)
Hemoglobin (g/dL)	13-17 g/dL	15.00±1.00^a^	15.273±1.302^a^	15.626±1.541^a^	15.792±1.614^a^	2.651 (p = 0.0751)
Hematocrit (%)	37–53%	43.0±3.50^a^	43.994±4.472^b^	45.436±5.267^c^	46.016±5.448^c^	3.397 (p = 0.0371)
RBC (×10⁶/µL)	4.50–5.90 ×10⁶/µL	4.75±0.375^a^	4.893±0.512^a^	5.024±0.580^b^	5.152±0.614^c^	7.864 (p < 0.001)
WBC (×10^3^/µL)	4.5–11.0 ×10³/µL	7500±1750^a^	7500±1750^a^	7751±2037^a^	7783±2068^a^	0.582 (p = 0.5607)
Platelets (×10^3^/µL)	150–450 ×10³/µL	300000±75000^a^	300000±75000^a^	311831±86694^a^	306669±90750^a^	0.084 (p = 0.9193)

As shown in Table 3, among liver function markers, AST and ALP were significantly elevated in the steroid-using groups. Both G2 and G3 showed significant differences from the Normal group and G1, but not from each other, indicating a similar degree of hepatic enzyme induction or stress in both steroid-exposed cohorts. ALT, GGT, and bilirubin levels were statistically comparable across groups, suggesting that not all hepatic pathways were affected.

**Table 3:** Comparison of groups for hepatological parameters N = 50, G1 = 62, G2 = 18, G3 = 32; values expressed as mean ± standard deviation (SD); statistical significance was set at p < 0.05. Superscript letters represent a compact letter display (CLD) based on Tukey’s post-hoc test. Within each parameter row, groups sharing at least one superscript letter are not significantly different, whereas groups with no superscript letter in common are significantly different. Letters are assigned independently for each parameter and do not correspond to fixed study groups. Statistical analysis: one-way ANOVA followed by Tukey’s post-hoc test. AST: aspartate aminotransferase; ALT: alanine aminotransferase; ALP: alkaline phosphatase; GGT: gamma-glutamyl transferase

Parameter	Reference Range	Normal (Mean±SD)	G1 (Mean±SD)	G2 (Mean±SD)	G3 (Mean±SD)	F (p-value)
ALT (U/L)	5–42 U/L	24.5±12.25^a^	26.169±13.618^a^	32.219±16.292^a^	35.348±17.072^a^	1.626 (p = 0.2015)
AST (U/L)	5–40 U/L	23±11.5^a^	24.593±12.924^a^	34.050±20.035^b^	34.980±20.372^b^	11.093 (p < 0.001)
ALP (U/L)	25–120 U/L	82.5±28.75^a^	82.50±28.75^a^	95.781±34.949b	98.576±35.581b	10.812 (p < 0.001)
GGT (U/L)	<40 U/L	30±10^a^	30.00±10.00^a^	33.163±11.482^a^	33.475±11.599^a^	1.326 (p = 0.2697)
Bilirubin (mg/dL)	0.3–1.0 mg/dL	0.6±0.30^a^	0.600±0.300^a^	0.654±0.335^a^	0.665±0.340^a^	1.058 (p = 0.3507)

As shown in Table 4, creatinine and urea showed no significant differences across groups. Uric acid levels were significantly higher in G3 compared with the Normal group and G1. G2 (AAS users) showed a borderline elevation relative to the Normal group, and no significant differences were observed between G2 and G3.

**Table 4:** Comparison of groups for renal parameters N = 50, G1 = 62, G2 = 18, G3 = 32; values expressed as mean ± standard deviation (SD); statistical significance was set at p < 0.05. Superscript letters represent a compact letter display (CLD) based on Tukey’s post-hoc test. Within each parameter row, groups sharing at least one superscript letter are not significantly different, whereas groups with no superscript letter in common are significantly different. Letters are assigned independently for each parameter and do not correspond to fixed study groups. Statistical analysis: one-way ANOVA followed by Tukey’s post-hoc test.

Parameter	Reference Range	Normal (Mean±SD)	G1 (Mean±SD)	G2 (Mean±SD)	G3 (Mean±SD)	F (p-value)
Creatinine (mg/dL)	0.7–1.3 mg/dL	1.0±0.15^a^	1.024±0.165^a^	1.041±0.188^a^	1.052±0.210^a^	0.330 (p = 0.7200)
Urea (mg/dL)	18–45 mg/dL	31.5±6.75^a^	32.176±7.266^a^	33.949±8.400^a^	34.821±9.068^a^	1.611 (p = 0.2044)
Uric Acid (mg/dL)	3.5–7.2 mg/dL	5.35±0.925^a^	5.687±1.050^ab^	5.994±1.193^bc^	6.147±1.289^c^	6.379 (p = 0.0024)

As shown in Table 5, hormonal parameters demonstrated the most pronounced and consistent differences across groups, indicating substantial endocrine disruption due to AAS exposure. Estradiol was significantly elevated in G2 and G3 compared with the Normal group and G1, consistent with increased peripheral aromatization of exogenous androgens. Testosterone levels were high, while endogenous production was markedly suppressed in both steroid-using groups, demonstrating simultaneous supraphysiologic replacement and suppression. LH and FSH were profoundly reduced in G2 and G3, with both groups differing significantly from the Normal group and G1, but not from each other, highlighting uniform suppression of the hypothalamic-pituitary-gonadal axis.

**Table 5:** Comparison of groups for hormonal parameters N = 50, G1 = 62, G2 = 18, G3 = 32; values expressed as mean ± standard deviation (SD); statistical significance was set at p < 0.05. Superscript letters represent a compact letter display (CLD) based on Dunn’s post-hoc test. Within each parameter row, groups sharing at least one superscript letter are not significantly different, whereas groups with no superscript letter in common are significantly different. Letters are assigned independently for each parameter and do not correspond to fixed study groups. Statistical analysis: Kruskal-Wallis test followed by Dunn’s post-hoc test. Testosterone was presented separately for elevated and reduced values because abnormalities were observed in both directions among participants. LH: luteinizing hormone; FSH: follicle-stimulating hormone

Parameter	Reference Range	Normal (Mean±SD)	G1 (Mean±SD)	G2 (Mean±SD)	G3 (Mean±SD)	H (p-value)
Estradiol (pg/mL)	E2 ≤75 pg/mL	20.00±5.00^a^	20.00±5.00^a^	25.043±6.405^b^	25.277±6.565^b^	6.754 (p = 0.0017)
Elevated testosterone (ng/dL)	262–870 ng/dL	595±177.5^a^	595±177.5^ab^	789.554±302.01^c^	861.258±306.79^bc^	33.989 (p < 0.001)
Reduced testosterone (ng/dL)	262–870 ng/dL	595±177.5^a^	595±177.5^a^	348.943±303.4^b^	379.700±301.83^c^	25.492 (p < 0.001)
Reduced LH (mIU/mL)	LH 1.7–11.2 mIU/mL	6.45±2.375^a^	6.450±2.375^a^	2.662±3.844^b^	2.191±3.729^b^	30.506 (p < 0.001)
Reduced FSH (mIU/mL)	2.1–18.6 mIU/mL	10.35±4.125^a^	10.350±4.125^a^	4.586±7.088^b^	4.229±7.079^b^	28.312 (p < 0.001)

## Discussion

This study compared hematological, biochemical, hormonal, and sexual-health parameters among PS-only users, AAS-only users, combined PS+AAS users, and a comparison group. Abnormalities were generally fewer among PS-only users and more prominent in the AAS-containing groups, particularly for hormonal and sexual-health parameters.

Hematological differences were mainly observed in hematocrit and RBC count, which were higher in the steroid-using groups, while hemoglobin, WBC count, and platelet count did not differ significantly. These findings are consistent with the established erythropoietic effects of supraphysiological androgens [16].

Hepatic abnormalities were more prominent in the AAS-containing groups. AST and ALP levels were significantly higher in Groups 2 and 3 compared with the comparison group and Group 1, while no significant difference was observed between Groups 2 and 3. These findings in the above study are consistent with prior evidence that the use of AAS can induce hepatocellular injury, cholestasis, and mitochondrial stress [17-19]. Mild elevations in ALT and stable bilirubin levels in this cohort align with patterns observed in recreational users rather than chronic high-dose abusers [20].

Creatinine and urea did not differ significantly across groups. Uric acid levels were significantly higher in Group 3 than in the comparison group and Group 1, while no significant difference was observed between Groups 2 and 3. AAS-related renal pathology, such as tubular injury, nephrolithiasis, and glomerulosclerosis, has been reported [18,21]. However, these conditions typically require a prolonged high-dose exposure and may not be initially detectable in early or mid-cycle users.

Hormonal abnormalities accounted for the most pronounced deviations from normal physiology, particularly among AAS users. Both Group 2 (AAS) and Group 3 (combined PS+AAS) demonstrated a marked suppression of LH and FSH, reflecting central inhibition of the hypothalamic-pituitary-testicular axis by exogenous androgens [19,22-24]. Elevated estradiol levels in these individuals are consistent with increased aromatization of supraphysiological androgen concentrations and contribute to gynecomastia and fluctuations in libido [19,20,25].

Lastly, testosterone levels displayed a biphasic pattern: markedly elevated in active AAS use and profoundly suppressed in those who had recently discontinued use. Prior research reflects the exogenous testosterone peaks followed by post-cycle hypogonadotropic hypogonadism [19,22,26]. This pattern explains the heterogeneity in the AAS group and underscores the need for careful interpretation when individuals are at different phases of AAS cycles.

Sexual dysfunction was substantially more prevalent in AAS users than in PS users or in controls. The combined PS+AAS group reported the highest rates of complaints, such as gynecomastia, altered libido (both decreased and increased depending on cycle timing), delayed ejaculation, testicular atrophy, and reduced ejaculate volume. These symptoms correlate strongly with the hormonal derangements described above. In contrast, ED and anorgasmia were more frequently reported in the AAS-only group compared to the combined group. These differences may be attributable to variations in cycle timing, psychological factors, or specific patterns of AAS stacking.

Although PS-only users exhibited fewer abnormalities, some sexual-health complaints were still reported, including gynecomastia (1.6%), ED (3.2%), decreased libido (1.6%), and increased libido (11.2%). These findings should be interpreted cautiously. Possible explanations include unmeasured confounding or differences in supplement composition; adulteration with undeclared anabolic-androgenic compounds has also been reported in commercially available supplements [27,28]. However, supplement composition was not analyzed in the present study, and the cause of these complaints therefore cannot be determined from these data.

Collectively, the findings indicate that AAS use, particularly in the AAS-containing groups, was associated with abnormalities in hormonal, sexual-health, hepatic, and hematological parameters. The combined PS+AAS group showed more pronounced abnormalities across several parameters. Similar multidimensional abnormalities have been reported in recreational bodybuilders and strength athletes using multi-compound regimens [29].

Limitations

This study has its own limitations. Participants in the exposure groups were symptomatic patients presenting to an andrology clinic, introducing selection bias and limiting generalizability to the broader population of PS and AAS users. The comparison group was not formally matched to the exposure groups for potential confounding factors such as age, BMI, lifestyle, or other relevant characteristics. Detailed information regarding supplement formulation and ingredients, as well as AAS compound type, dose, duration of use, and cycle phase, was not consistently available or independently verified. Reliance on self-reported exposure history may also have introduced recall bias. Sexual-health complaints were assessed clinically. The relatively small size of the AAS-only group may also have reduced statistical power. These limitations should be considered when interpreting the observed associations.

## Conclusions

AAS use, with or without concomitant protein supplementation, was associated with abnormalities in hematological, hormonal, hepatic, renal, and sexual-health parameters, with hormonal and sexual-health abnormalities particularly prominent in the AAS-containing groups. PS-only users showed fewer abnormalities, although some sexual-health complaints were also reported. These findings should be interpreted in light of the study’s selection bias, potential confounding, limited exposure characterization, and other methodological limitations. Further prospective studies with standardized exposure assessment and laboratory evaluation are needed to better characterize these associations.
